# Long Non-Coding RNAs in the Cell Fate Determination of Neoplastic Thymic Epithelial Cells

**DOI:** 10.3389/fimmu.2022.867181

**Published:** 2022-04-22

**Authors:** Alessia Iaiza, Claudia Tito, Federica Ganci, Andrea Sacconi, Enzo Gallo, Silvia Masciarelli, Giulia Fontemaggi, Alessandro Fatica, Enrico Melis, Vincenzo Petrozza, Federico Venuta, Mirella Marino, Giovanni Blandino, Francesco Fazi

**Affiliations:** ^1^ Department of Anatomical, Histological, Forensic and Orthopedic Sciences, Section of Histology and Medical Embryology, Sapienza University of Rome, Rome, Italy; ^2^ Oncogenomic and Epigenetic Unit, IRCCS Regina Elena National Cancer Institute, Rome, Italy; ^3^ Department of Pathology, IRCCS Regina Elena National Cancer Institute, Rome, Italy; ^4^ Department of Life Science and Public Health, Histology and Embryology Unit, Catholic University of the Sacred Hearth, Rome, Italy; ^5^ Department of Biology and Biotechnology ‘Charles Darwin’, Sapienza University of Rome, Rome, Italy; ^6^ Thoracic Surgery, IRCCS Regina Elena National Cancer Institute, Rome, Italy; ^7^ Pathology Unit, ICOT, Department of Medico-Surgical Sciences and Biotechnologies, Sapienza University of Rome, Latina, Italy; ^8^ Department of Thoracic Surgery, Sapienza University of Rome, Rome, Italy

**Keywords:** thymoma, thymic carcinoma, thymic epithelial tumors (TETs), ncRNAs (non coding RNAs), miRNA - microRNA, lncRNA - long noncoding RNA, MALAT1, myasthenia gravis

## Abstract

Thymic Epithelial Tumors (TETs) arise from epithelial cells of the thymus and are very rare neoplasms comprising Thymoma, Thymic carcinoma, and Thymic Neuroendocrine tumors that still require in-depth molecular characterization. Long non-coding RNAs (lncRNAs) are emerging as relevant gene expression modulators involved in the deregulation of several networks in almost all types of human cancer, including TETs. LncRNAs act at different control levels in the regulation of gene expression, from transcription to translation, and modulate several pathways relevant to cell fate determination under normal and pathological conditions. The activity of lncRNAs is strongly dependent on their expression, localization, and post-transcriptional modifications. Starting from our recently published studies, this review focuses on the involvement of lncRNAs in the acquisition of malignant traits by neoplastic thymic epithelial cells, and describes the possible use of these molecules as targets for the design of novel therapeutic approaches specific for TET. Furthermore, the involvement of lncRNAs in myasthenia gravis (MG)-related thymoma, which is still under investigation, is discussed.

## Introduction

The thymus is the primary lymphoid organ located at the level of the anterior mediastinum. The thymus plays an essential role in educating and enabling the maturation of prelymphocytes into mature T lymphocytes, which are involved in the adaptive immune response. Thymic epithelial-reticular cells (TECs) play a major role in the maturation of T lymphocytes. However, TECs may undergo neoplastic transformation, resulting in certain types of tumors, such as thymic epithelial tumors (TETs) (1).

TETs are relatively rare neoplasms in middle-aged or elderly adults that represent 0.2-1.5% of all cancers and comprise thymoma, thymic carcinoma (TC), and thymic neuroendocrine tumors (2). TETs are characterized by wide variability and heterogeneity in their malignant behavior. In 30% of cases, TETs are asymptomatic; however, in 40% of cases, local symptoms, such as chest pain, cough, dyspnea, and hoarseness, are displayed while in the remaining 30% of cases, systemic symptoms, with superior vena cava syndrome (SVC), and in the most aggressive forms, weight loss, ensue. To date, the etiology of TETs has not been established and the risk factors remain unclear (3).

TETs classification has remained controversial subject for many years. After different classification approaches [Bernatz et al. in 1961, Rosai and Levine in 1976, Marino and Muller- Hermelink, in 1985 (4)], Rosai and Sobin published a new classification in the World Health Organization (WHO) series in 1999, dividing thymic tumors into three major subgroups based on the morphology of epithelial cells and the percentage of epithelial and lymphocyte populations: type A, type B, and type C (thymic carcinoma). Type A thymomas are tumors with a component of spindle-oval EC but lack lymphocytes, whereas type B thymomas are characterized by large EC with dendritic or plump (epithelioid) morphology, forming networks where lymphocytes are attracted. Notably, the combination of these two morphologies has been designated as type AB (5). Thymic carcinoma (type C) is a rare malignancy, representing less than 1% of thymic tumors, and is characterized by cytological atypia, more aggressive behavior, and local and distant metastases (liver, lymph nodes, or bones) (6).

Recently, a new WHO classification of thoracic cancers was established, which includes new diagnostic criteria and rare entities, such as hyalinizing clear cell carcinoma (7).

Interestingly, thymoma is strongly associated with various paraneoplastic syndromes (PNS), such as myasthenia gravis (MG), red cell aplasia, polymyositis, systemic lupus erythematosus, Cushing syndrome, and syndrome of inappropriate antidiuretic hormone secretion (8).

Thymomas are associated with MG in 30-50% of cases, and thymoma occurs in 10-15% of cases of MG (9). MG (myos = muscle, asthenos = weakness, gravis = severe) is an autoimmune disease that affects the neuromuscular junction (NMJ) of the skeletal muscle, causing muscle weakness of different severity, several complications such as myasthenia crisis, and in some cases, acute respiratory paralysis (10–13). MG can develop at any age, particularly in young women (>30 years) and older men (>60 years) (14).

Thymoma-associated myasthenia gravis (TAMG) is frequently reported in adults and is characterized by alterations in thymus function (14). The association between MG and thymoma is due to the dysregulation of positive and negative selection of T cells in the thymus (15). For example, in cortical thymoma, the lack of medullary epithelial cell function and defects in the autoimmune regulator complex (AIRE), which is responsible for negative selection, leads to the production of autoreactive T cells specific for acetylcholine receptors (AChR) that are exported to the periphery, where autoreactive T cells stimulate and activate B cells to produce antibodies against AChR (14, 16). Although the required treatment for thymoma-associated MG is tumor removal, remission is not inevitable. Moreover, removal of thymoma in non-myasthenic patients does not prevent the subsequent onset of MG. Patients with thymoma have been observed to develop antibodies against AChR and symptoms of myasthenia after the resection of the tumor (17).

## Molecular Pathways in TETs and MG

Although the etiology of TETs is still poorly understood, advanced next-generation sequencing (NGS) techniques have recently allowed the mapping of gene mutations and epigenetic alterations occurring in thymic tumors.

One of the most frequently mutated genes in TETs is *GTF2I*, whose mutation is specifically associated with types A and AB (78%), but is less frequent in more aggressive histological types, such as thymic carcinomas (8%) (18). The overexpression of EGFR and HER2 and mutations in KIT, IGR-1, and neurotrophin receptors have been recently demonstrated in some cases, as reviewed by Scorsetti et al. (6). Gain-of-function mutations in HRAS and NRAS and loss-of-function mutations in TP53 are less common, but are considered founder mutations (19).

Thymic carcinoma is characterized by loss of chromosome 16q, mutations in epigenetic regulatory genes (*BAP1*, *ASXL1*, *SETD2*, *SMARCA4*, *TET2*, *DNMT3*A, and *WT1*), and anti-apoptotic genes (*BCL2* copy number gains) (8, 20).

Notably, gene expression profiling in TGCA study revealed four molecular subtypes, represented respectively by type B (subtype 1), TC (subtype 2), AB (subtype 3), and a mix of types A and AB (subtype 4). TGCA study also revealed four distinct molecular clusters using PARADIGM analysis. In particular, the upregulation of TP53 and downregulation of oncogenes, such as MYC/Max, MYB, and FOXM, characterize the A-like cluster, while the downregulation of TP53 and upregulation of MYC/Max, MYB, FOXM1, and E2F1 in AB-, B-, and C-like clusters are consistent with the high aggressiveness of B3 and TC tumors. Furthermore, this study highlighted that types A, AB, B, and TC are not a continuum of diseases, but are instead distinct biological entities (5, 19), Recently, metaplastic thymomas were reported to harbor the YAP1-MAML2 translocation, whereas 6% of pretreated types B2 and B3 and a combined TC and B3 thymoma (but not in thymoma and “pure” TCs) may be associated with KMT2A-MAML2 translocation (7).

Alterations in inflammatory and thymus function, which occur in thymic neoplasms, and mutations in the *AIRE* gene locus promote the development of autoimmune diseases, such as MG (16). EOMG is associated with HLA-DR3, HLA-B8, and other autoimmune risk genes, whereas LOMG is weakly associated with HLA-DR2, HLA-B7, and mLA-DR-B1*15:0 (14). Aneuploidy and intratumoral overexpression of genes that have a similar sequence to autoimmune targets (CHRNA1, RYR3, and NEFM) are common in patients (19).

In addition to mutations in protein-coding genes, alterations in ncRNA molecules have been reported to significantly impact the initiation, progression, and response of TETs and MG to therapy, as described in the next section.

## Identification of ncRNAs in TETs and MG

Despite being considered “junk” for a long time, ncRNAs have emerged as functionally relevant in nearly all physiological and pathological cellular processes (21, 22). These new discoveries have been aided by powerful high-throughput approaches, such as next-generation sequencing (NGS), transcriptome studies, molecular network analyses, and artificial intelligence-guided prediction of ncRNA function (23).

ncRNAs play a role in many biological, physiological, and developmental processes, including several diseases and tumors. ncRNAs are produced by transcription from different genomic regions and post-transcriptional maturation and modification. ncRNAs can be divided into two classes according to their length (24): small non-coding RNAs (ncRNAs) and lncRNAs (usually > 200 nt).

In recent years, ncRNAs have been found to play an important role in gene regulation at different levels. Several studies have demonstrated the involvement of ncRNAs in transcriptional regulation, RNA maturation, chromatin remodeling, post-transcriptional RNA regulation, and modification (25). Dysregulation of ncRNAs is involved in many human diseases and in tumor initiation and progression (26). Regulatory ncRNAs can be divided into two classes: circular and linear.

Circular RNAs (circRNAs) are a class of covalently closed RNA molecules and are thus more stable than linear RNAs. The expression patterns of circRNAs are cell type-, tissue-, and developmental stage-specific (27, 28). Depending on their localization, circRNAs can exert different functions, including acting as microRNA (miRNA) sponges, modulating the activity of RNA-binding proteins (RBPs), or acting as protein scaffolds, which can be translated into polypeptides owing to the presence of internal ribosome entry sites (IRES) or m^6^A modifications (27, 29). Many circRNAs are differentially expressed in several cancer types compared with their untransformed counterparts and are related to tumor growth, metastasis, and therapy resistance (30, 31). Interestingly, several studies have demonstrated that circRNAs are differentially expressed in thymoma and MG (32, 33), and have highlighted their important role as biomarkers for the diagnosis of this disease (34).

Another major group of regulatory ncRNAs is represented by linear ncRNAs, comprising small ncRNA (18-200 nt) and lncRNAs (>200 nt) (35).

Among small ncRNAs, miRNAs play a predominant role in post-transcriptional regulation, binding to specific mRNA targets and causing their degradation or translation inhibition. miRNAs are single-stranded RNAs with an average length of 22 nucleotides that are derived from hairpin-structured precursors (36). Several miRNAs are altered in different human diseases, including cancer. The roles of miRNAs in thymus differentiation, development, and involution have been extensively described by Cron et al. (37). Moreover, their relevance in the treatment, diagnosis, and prognosis of TETs and MG is emerging (38).

Several research groups have identified miRNAs that are differentially expressed between thymic tumors and normal samples, as well as between thymic carcinoma and thymoma and histotype classes (39–41). In particular, Bellissimo et al. found that miR-145-5p was epigenetically downregulated in thymic carcinoma cells (42), confirming its well-known tumor suppressor role (43). Similar to circRNAs, miRNAs are excellent biomarkers for cancer diagnosis found in the serum of patients with TETs and MG (44–49).

## LncRNAs in TETs

Although miRNAs represent one of the most studied biomarkers involved in thymic tumorigenesis among all ncRNA classes, the study of lncRNAs is becoming more relevant. Based on their localization, lncRNAs can bind to genes, transcripts, miRNAs, and proteins that regulate different cellular processes, such as gene expression, transcription, and post-transcriptional regulation, through different mechanisms of action. Further, lncRNAs can act as guides of chromatin modification complexes, including DNA methyltransferase and histone-modifying enzymes, on specific genomic loci that induce activation or inhibition of target genes in *cis* or *trans* (50); function as decoys for transcription factors and other effectors, impairing their regulatory activity (50); serve as modular scaffolds, and bind and drive two or more physically distant proteins into specific genomic regions to regulate gene expression (51); act as sponges of miRNAs, sequestering them and preventing their ability to promote degradation or repression of target genes (52, 53); and influence splicing (54) and the stability of mRNAs, regulating their post-transcriptional expression (55). Therefore, according to their mechanisms of action, lncRNAs are involved in the regulation of various biological processes, including cellular survival, proliferation, differentiation, apoptosis, invasion, and metastasis. Consequently, aberrant lncRNA expression is tightly correlated with cancer development (56).

### Deregulation of lncRNAs in TETs

**Figure 1:**
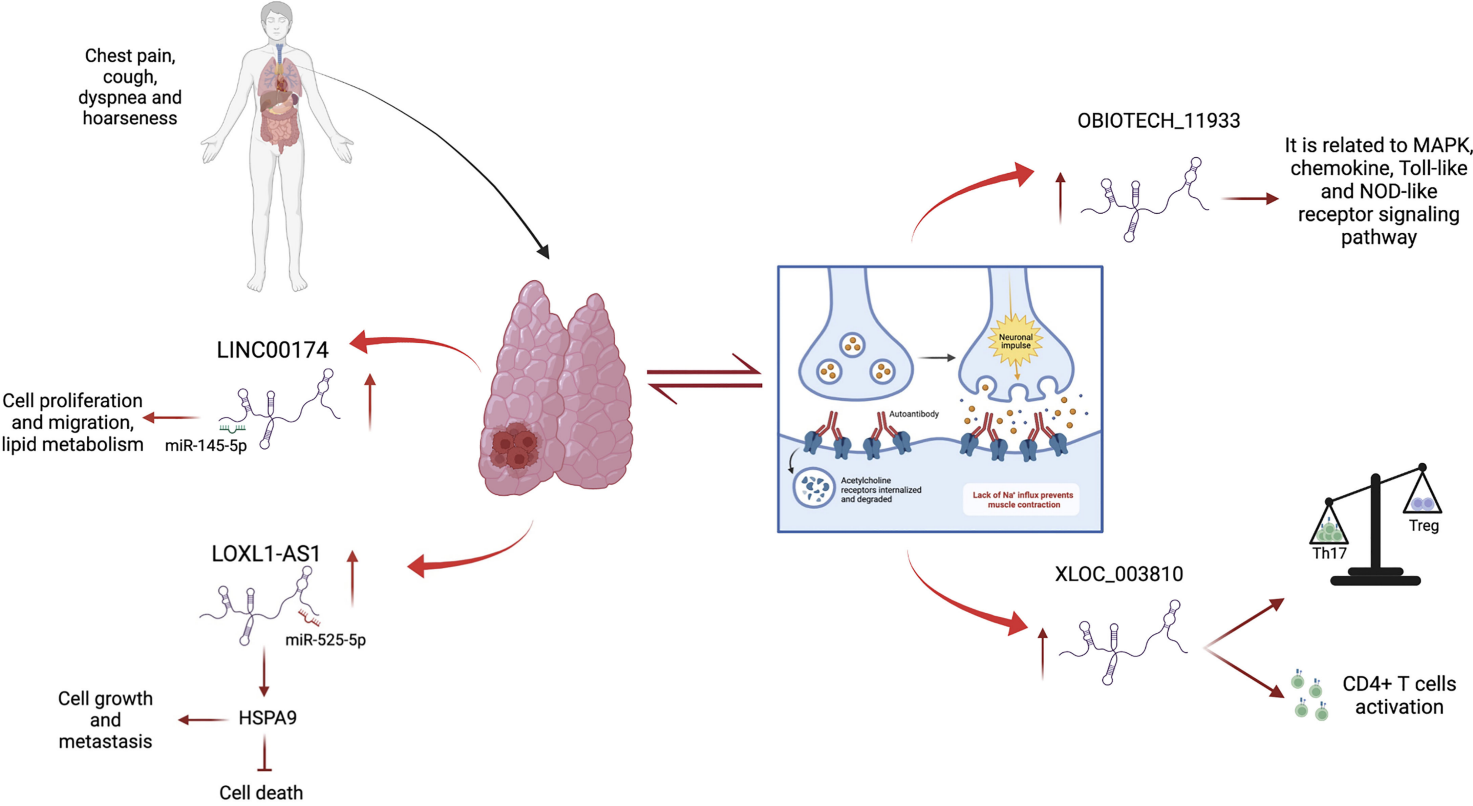
Contribution of lncRNAs in TETs and MG. LINC00174 and LOXL1-AS1 are up-regulated in thymoma and thymic carcinoma, where they act as sponges of miR-145-5p and miR-525-5p, respectively, promoting cell growth, metastasis, migration, and lipid metabolism and inhibiting cell death. LncRNAs are deregulated in MG, an autoimmune disease strongly associated with thymoma. Oebiotech_1193 and XLOC_003810 are overexpressed in MG patients and are involved in inflammatory pathways.

Similar to many types of human tumors, such as breast cancer (57), lung cancer (58), colorectal carcinoma (59), ovarian cancer (60) and prostate cancer (61), dysregulated lncRNAs in TETs may contribute to tumor onset and progression. Different studies have identified a large spectrum of lncRNAs in thymic epithelial tumors using high-throughput sequencing technologies. In this context, most of the identified lncRNAs act as miRNA sponges, playing oncogenic or tumor suppressor roles. An example of this type of regulation is represented by lncRNA LOXL1-AS1, miR‐525-5p, and the HSPA9 gene network. Data from TCGA study showed that high expression of LOXL1-AS1 and downregulation of miR-525-5p correlated with poor prognosis in TET (62). Similar to other cancer types (63, 64) in thymic tumors, miR-525-5p acts as a tumor suppressor, inhibiting cell growth and invasion, and inducing apoptosis by repressing the target gene, *HSPA9*. *HSPA9* is upregulated in thymoma and thymic carcinoma and correlated with poor patient survival. The positive association between LOXL1-AS1 and HSPA9, which is consistent with the downregulation of miR-525-5p, was confirmed by *in vitro* experiments. The silencing of LOXL1-AS1 promotes thymic tumor progression by acting as a sponge of miR-525-5p and increasing the expression of HSPA9 (62). Similar to LOXL1-AS1, another network is represented by the interaction between lncRNA LINC00174, miR-145-5p, and miR-145-5p predicted target genes involved in thymic tumorigenesis (65). In this study, the upregulation of lncRNA LINC00174 in frozen tissue samples of thymoma compared to its normal counterparts was identified. LINC00174 is negatively associated with miR-145-5p, a well-known tumor suppressor of miRNA downregulation in TETs (39, 42), and positively correlated with miR-145-5p predicted targets (**Figure 1**). The inhibition or overexpression of miR-145-5p modulates LINC00174 expression and its associated genes (65). Notably, the poor prognosis of TET patients, characterized by high expression of LINC00174 and its associated genes and low expression of miR-145-5p, suggests an oncogenic role of LINC00174 in TETs. According to these data, LINC00174 silencing impairs cell growth and proliferation, cell migration, and lipid metabolism. Similar to LINC00174, MALAT1 can act as a sponge for miR-145-5p in TET. MALAT1 is a well-known oncogenic lncRNA that regulates different biological processes, such as cell proliferation, apoptosis, angiogenesis, invasion, and metastasis, and contributes to cancer development (66–68). Using a luciferase assay, the interaction between miR-145-5p and MALAT1 was demonstrated in the thymic cancer cell line, IU-TAB1. In this thymic tumor context, the downregulation of MALAT1 increased miR-145-5p expression and led to a reduction in cell proliferation and an increase in the apoptosis rate compared to that observed in the control. Additionally, the combination of MALAT1 silencing and miR-145-5p overexpression induces a synergistic effect, suggesting that MALAT1 may regulate the thymic cancer phenotype by inhibiting miR-145-5p (69). Recently, the expression of MALAT1 has been a focus in our studies where the relationship between lncRNA MALAT1 and METTL3, a methyltransferase enzyme that catalyzes the N6-methyladenosine (m^6^A) modification, were described in the thymic carcinoma cell line, TC1889. Of note, the expression of lncRNAs can be regulated by m^6^A modifications (70), and the downregulation of METTL3 leads to increased localization of MALAT1 in nuclear speckles and decreased m^6^A modification of MALAT1 lncRNA (71), which probably impinges on its functional activity. In the past, we observed a similar delocalization of MALAT1 in nuclear speckles with consequent altered splicing (72) due to the presence of mutant p53 protein in breast cancer cells. Another interesting lncRNA-miRNA-target network in the control of thymoma progression was reported by Yang et al. (73). The lncRNA RP11-424C20.2 regulates the expression of the *UHRF1* gene (ubiquitin-like containing PHD ring finger 1) by sponging miR-378a-3p; the RP11-424C20.2/UHRF1 axis is strongly associated with a better outcome in thymoma patients, which is related to the different types of infiltrating immune cells, such as B cells, macrophages, CD8+ and CD4+ cells, neutrophils, and dendritic cells. The role of UHRF1 is well established; it is an epigenetic modifier that regulates immune infiltration and the tumor immune microenvironment through its interaction with DNA histone deacetylase genes (73, 74). Therefore, RP11-424C20.2 expression can influence the prognosis of patients with thymoma by regulating the expression of UHRF1 *via* miR-378a-3p sponging (73).

Owing to this recent evidence, the identification of altered lncRNAs in TETs and the characterization of their role in the promotion of tumorigenesis could provide new potential therapeutic targets relevant for the treatment of TETs.

### LncRNAs as a Predictive Factor of Patient Prognosis in TETs

Studies on the profiles of lncRNAs expressed in thymoma tissue samples have revealed that altered expression of specific lncRNAs may correlate with overall or disease-free survival. For example, Su et al. (75) identified a panel of lncRNAs that predicts the recurrence of thymic epithelial tumors. They analyzed a cohort of 114 TET patients from TCGA study and identified four lncRNAs, ADAMTS9-AS1, HSD52, LINC00968, and LINC01697, which are significantly related to recurrence-free survival (RFS). These lncRNAs can be used to divide TET patients into high-risk and low-risk groups, respectively, with shorter and longer RFS. Based on ROC analysis, these lncRNAs represent a better prognostic model for the RFS of patients than the WHO classification and Masaoka stage. Although these lncRNAs constitute a good factor of discrimination between different TETs subtypes and their associated stages, the trial had some limitations: few samples from TCGA, absence of evidence of their predictive power in other types of cancer, and biological characterization of their role in TETs (75). Furthermore, the altered expression of ADAMTS9-AS1 (one of the four RFS-related lncRNAs) with five other lncRNAs, namely AFAP1-AS1, LINC00324, VLDLR-AS1, LINC00968, and NEAT1, was detected in another study by RNA-seq and profiling expression analysis in 25 thymoma patients and 25 healthy individuals (76). These lncRNAs are involved in the development of different types of cancers and regulate several biological processes and molecular pathways. For example, ADAMTS9-AS1 induces cell migration and proliferation in colorectal carcinoma, affecting β-catenin expression (77); LINC00968 reduces drug resistance and invasion of tumor cells in breast cancer (78); AFAP1-AS1 increases epithelial-mesenchymal transition by impairing RhoC, ROCK1, p38MAPK, and Twist1 signaling pathways in osteosarcoma (79); LINC00324 inhibits the NOTCH pathway, regulating apoptosis and cell proliferation in papillary thyroid cancer (80); VLDLR- AS1 modulates the expression of genes involved in fat loss in cancer cachexia, acting as a sponge of hsa-miR-1224-3p (81) and finally, NEAT1, by sponging miR-193b-3p, activates cyclin D, promoting cell proliferation in cervical cancer (82). Notably, the differential expression of these lncRNAs in TETs affects the disease-free survival of patients. In particular, the high expression of ADAMTS9-AS1 and low expression of LINC00324 are correlated with the worst prognosis of patients. Similar to other types of cancer, the expression of these lncRNAs was found to be correlated with the deregulation of miRNA clusters and target genes involved in the regulation of tumorigenic signaling pathways, including PI3K/Akt, FoxO, HIF-1, and Notch, supporting their oncogenic role in the tumorigenesis process (76). Moreover, Gong et al. found that AFAP1-AS1, LINC00324, and VLDLR-AS1 were associated with the RFS of patients with TETs (83).

According to these data, bioinformatic analysis performed for different types of TETs (A, B, AB, and TC) revealed that different competitive endogenous RNA (ceRNA) networks were significantly associated with the overall survival of individuals. The two most important lncRNAs in this ceRNA network were LINC00665 and NR2F1-AS1. The association between their expression and patient prognosis aligns with their biological function (84). LINC00665 binds to mRNAs MYO10 and WASF3 through the miRNAs, hsa-miR-140 and hsa-miR-3199. LINC00665 is upregulated in lung cancer, regulates cellular proliferation and invasive ability in lung adenocarcinoma, and is a predictive factor of this tumor (85). NR2F1-AS1 can indirectly interact with FBN1, GALNT16, HAND2, and MCAM through miR-140, miR-139, and miR-141. NR2F1-AS1 leads to the impairment of osteosarcoma, acting as a sponge of miR-483-3p and increasing FOXA1 gene expression (86).

Based on these recent studies, profiling analysis of lncRNA expression can be used as a potential and innovative strategy for the detection and follow-up of thymic epithelial tumors (**Table 1**).

**Table 1:** LncRNAs deregulated in TETs.

lncRNAs	Expression	Biological function	Prognostic clinic value	References
**LOXL1-AS1**	Upregulated in thymoma and thymic carcinoma	LOXL1-AS1 acts as a sponge for miR-525-5p, increasing HSPA9 expression.	High levels of LOXL1-AS1 and HSPA9 are associated with poor prognosis	**Wang et al.** (62)
**LINC00174**	Upregulated in thymoma and thymic carcinoma	LINC00174 acts as a sponge for miR-145-5p	High levels of LINC00174 and low level of miR-145-5p are associated with poor prognosis	**Tito et al.** (65)
**MALAT1**	Upregulated in thymic carcinoma	MALAT1 acts as a sponge for miR-145-5p. MALAT1 localization is m^6^A-dependent and is involved in c-MYC induction	High levels of MALAT1 are associated with poor prognosis	**Tan et al.** (69) **Iaiza et al.** (71)
**RP11-424C20.2**	Upregulated in thymoma	RP11-424C20.2 acts as a sponge for miR-378a-3p, increasing UHRF1 expression	High levels of RP11-424C20.2 and UHRF1are associated with better prognosis	**Yang et al.** (73)
**AFAP1-AS1** **LINC00324** **VLDLR- AS1**	Upregulated in thymoma	They are involved in the regulation of cell proliferation	High levels of AFAP1-AS1 and low levels of LINC00324 and VLDLR- AS1 are associated with poor disease-free survival	**Ji et al.** (76)
**LINC00665**	Upregulated in thymoma	LINC00665 acts as a sponge for miR-140 and miR-3199, increasing MYO10 and WASF3	High levels of LINC00665 are associated with poor overall survival	**Chen et al.** (84)
**NR2F1-AS1**	Upregulated in thymoma	NR2F1-AS1 acts as a sponge for miR-140, miR-139 and miR-141, increasing FBN1, GALNT16, HAND2, and MCAM expression	High levels of NR2F1-AS1 are associated with poor overall survival	**Chen et al.** (84)

## LncRNAs in MG

**Figure 2:**
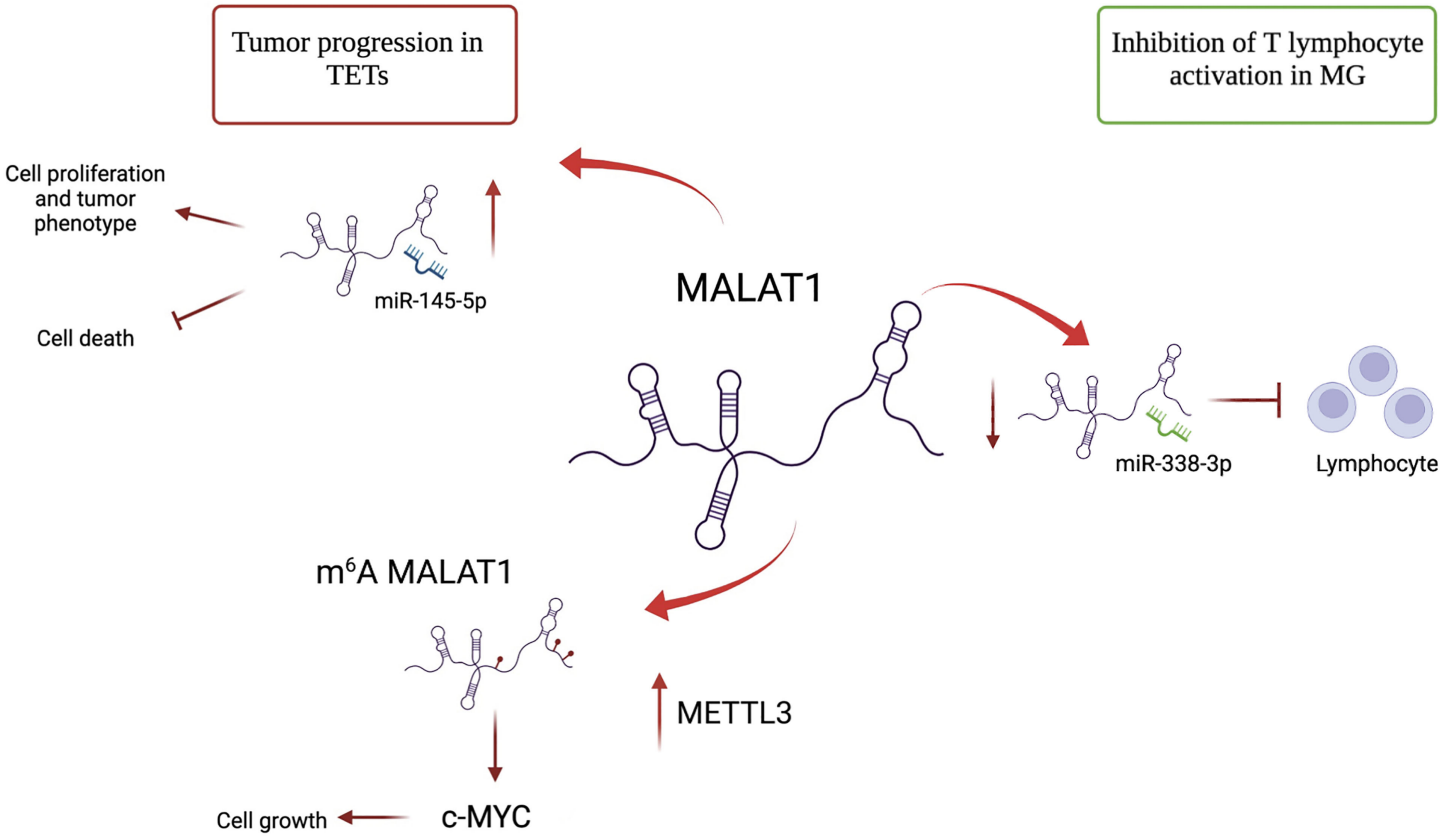
Different roles of lncRNA MALAT1 in TETs and MG. MALAT1 has an oncogenic role in TETs. It promotes cell proliferation by acting as a sponge of miR-145-5p. MALAT1 is m^6^A-modified due to METTL3 overexpression in TET and induces c-MYC protein, further contributing to proliferation. In MG, MALAT1 plays a protective role by binding mir-338-3p and avoiding T lymphocyte activation.

The alteration of lncRNA expression could play a prominent role in distinguishing thymomatous and non-thymomatous MG and clarifying the molecular mechanisms underlying its pathogenesis. In this context, by using lncRNA and mRNAs microarray analyses, Luo et al. (87) identified an aberrant expression of different lncRNAs between MG patients with thymoma and healthy controls, and MG patients without thymoma and normal individuals. In the first case, lncRNAs upregulated in MG patients with thymoma were associated with different regulatory pathways that contribute to thymic cancer progression and immune cell proliferation, such as cell response to interferon-γ, positive regulation of cytokine production, chemokine receptor binding, and regulation of smooth muscle cell proliferation. In particular, the most upregulated lncRNA in MG patients with thymoma is Oebiotech_11933, an lncRNA related to the MAPK, chemokine, and Toll-like receptor signaling pathways (87–89). In the second case, although altered lncRNAs in MG patients without thymoma revealed their association with the same cellular pathways in MG patients with thymoma (i.e., positive regulation of cytokine production and chemokine receptor binding), they showed a lower association with cell response to interferon-γ. These data highlight that the discrimination between MG patients with or without thymoma may depend on the presence of altered lncRNAs involved in the regulation of IFN-γ expression (90). Additionally, these lncRNAs have been observed to function by regulating the transcription of genes in *cis* or *trans* (87). Consistent with this study, Ke et al. found another lncRNA, XLOC_003810, which is highly expressed in MG-associated thymoma patients, and revealed an increase in activated CD4+ T cells compared to that in control samples. *In vitro* experiments using thymic mononuclear cells demonstrated that the overexpression of XLOC_003810 leads to an increase in CD4+ T cells and production of the inflammatory cytokines IFN-γ, TNF-α, and IL-1β. In contrast, the downregulation of XLOC_003810 caused the opposite results. Consequently, as the activation of CD4+ T cells and inflammatory cytokines plays an important role in the development of thymoma-associated MG, XLOC_003810 lncRNA could contribute to the pathogenesis of these cellular pathways (91). The study by Niu et al. supports the role of XLOC_003810 in MG with thymoma. XLOC_003810 affects the balance between T helper 17 (Th17) and T regulatory cells (Tregs) (92). T helper cells are active in the adaptive immune response against antigens and pathogens, and Tregs have suppressive potential, preventing autoimmune diseases (93). The overexpression of XLOC_003810 leads to higher levels of Th17 cells than Tregs, which, on the contrary, increases upon silencing of this lncRNA. This association is also evident in MG-T patients and is characterized by an increase in CD4+ T cells and Th17 cells and a decrease in Treg cells (92). As the number of Tregs increases in MG-T patients upon immunosuppressive treatment (94) and the number of Th17 cells correlates with the severity of the disease (95), the alteration of XLOC_003810 expression could enhance the imbalance in the Th17/Treg ratio, favoring the pathogenetic mechanism. Moreover, the discrimination between patients with MG with or without thymoma is also determined by the different hypomethylation and hypermethylation levels associated with the aberrant expression of lncRNAs. The presence of DNA methylation sites has been observed in three immune-related lncRNAs, namely AC004943.1, FOXG1-AS1, and WT1-AS, in (MG-T) patients. DNA methylation is an epigenetic modification catalyzed by DNA methyltransferase enzymes that promote the silencing of gene expression. In this context, tissue samples of thymoma patients with MG are characterized by lower methylation levels of these lncRNAs than those without MG. Consequently, MG-T patients showed a higher expression of these immune-related lncRNAs that correlate with their involvement in pathogenesis, regulating different biological processes, such as transmission at the neuromuscular junctions, cell cycle, actin and Ras GTPase binding, and herpes simplex virus 1 infection associated with MG development (96). Although the lncRNA, MALAT1, is a known oncogene that promotes thymic cancer development, as described previously, it plays a protective role in MG (**Figure 2**). Compared to healthy individuals, lower expression of MALAT1 has been observed in MG patients, together with higher expression of miR-338-3p, an oncogenic miRNA that directly targets MSL2, a gene involved in chromatin organization and DNA damage response. A previous study revealed the interaction between MALAT1, miR-338-3p, and MSL2 by luciferase assay, demonstrating that the silencing of MALAT1 leads to an increase in miR-338-3p expression, reducing MSL2 protein levels (97). The downregulation of MALAT1 in MG patients suggests its involvement in the inhibition of T lymphocytes, suggesting that it could be a specific target for MG treatment. Finally, different lncRNAs are involved in the regulation of hydrolase, phosphorylase, and dephosphorylase enzyme activities, which affect the activation of T cells during selection in the thymus, promoting MG development (98).

Based on the evidence, a large spectrum of lncRNAs can regulate different signaling pathways that contribute to the development of associated MG-thymoma. As a result, they could be used as biomarkers to distinguish between two types of MG, namely MG with or without thymoma. Moreover, the data suggest the possible use of these pathogenesis-related molecules as therapeutic targets (**Table 2**).

**Table 2:** LncRNAs deregulated in Myasthenia Gravis.

lncRNAs	Expression	Biological function	Prognostic clinic value	References
**XLOC_003810**	Upregulated in myasthenia gravis associated with thymoma	XLOC_003810 increases CD4+ T cell activation and inflammatory cytokines, such as IFN-γ, TNF-α, and IL-1β XLOC_003810 regulates Th17/Treg balance	CD4+ T cells are activated, and inflammatory cytokines are significantly expressed in the thymic tissue of MG patients with thymoma. Positive correlation between XLOC_003810, which promotes the shift in Treg cells toward Th17 cells, and the clinical severity of the disease in MG-T patients.	**Hu et al.** (91) **Niu et al.** (92)
**AC004943.1** **FOXG1-AS1** **WT1-AS**	Upregulated in myasthenia gravis associated with thymoma	They are involved in the regulation of transmission of neuromuscular junctions, cell cycle, actin, Ras GTPase binding, and herpes simplex virus 1 infection.	The higher expression of these lncRNAs was correlated with a lower DNA methylation level and influence the prognosis of thymoma	**Zhuang et al.** (96)
**MALAT1**	Downregulated in myasthenia gravis associated with thymoma	MALAT1 acts as a sponge of miR-338-3p, reducing MSL2 expression levels	Low expression of MALAT1 in MG patients compared with controls, suggesting that it inhibited T lymphocyte activation and the protective effect in the pathogenesis of MG	**Kong et al.** (97)

## Concluding Remarks

Recent advances in next-generation sequencing technologies have enabled the study of the role of ncRNAs in the development and progression of cancer. Particularly, the aberrant expression of the most studied groups of ncRNAs, such as miRNAs, circRNAs, and lncRNAs, is associated with tumorigenesis, highlighting the role of ncRNAs as oncogenes or tumor suppressors. In this review, we sought to provide an overview of lncRNA regulation in the initiation and progression of TET and MG. Many lncRNAs identified in these diseases play an oncogenic role, acting as sponges of tumor suppressor miRNAs and consequently regulating many cellular pathways that contribute to the cancer phenotype. The identification of aberrant expression of lncRNAs and studies on their inhibition or overexpression allow us to understand their contribution to the thymic cancer phenotype and suggest specific targeted therapies. Various lncRNAs differentially expressed in tumor vs. normal tissues in patients with TET are potential powerful biomarkers for the detection and follow-up of diseases.

The role of the lncRNA, MALAT1, which has several opposing functions, is particularly intriguing. In thymoma and thymic carcinoma, MALAT1 regulates cell proliferation by acting as an miR-145-5p sponge and contributing to c-MYC induction, following its change in subnuclear localization due to METTL3 methylation. In contrast, MALAT1 has a protective role in MG, acting as an miRNA sponge and inhibiting T lymphocyte activation. Although several lncRNAs have been identified to date, the function and expression of many lncRNAs in TETs and MG pathogenesis and progression remain unclear. Therefore, further studies on these ncRNAs are necessary. Moreover, the development of novel lncRNA-directed therapeutic strategies could represent a promising and powerful approach for the management of TET and MG.

## Author Contributions

AI and CT designed and wrote the manuscript. SM, GF, MM, and FF reviewed and edited the manuscript. FG, AS, EG, AF, EM, VP, FV, and GB conceptualized the study and provided feedback regarding the content of the manuscript. All authors approved the final version of the manuscript.

## Funding

AIRC IG 2018 - ID. 21406 project, ‘Progetti Ateneo’ Sapienza University of Rome and PRIN 2017-Prot. 2017TATYMP_003 to FF.; AIRC IG 2018 - ID. 21434 Project to GF; and ‘Progetti Ateneo,’ Sapienza University of Rome to VP.

## Conflict of Interest

The authors declare that the research was conducted in the absence of any commercial or financial relationships that could be construed as a potential conflict of interest.

## Publisher’s Note

All claims expressed in this article are solely those of the authors and do not necessarily represent those of their affiliated organizations, or those of the publisher, the editors and the reviewers. Any product that may be evaluated in this article, or claim that may be made by its manufacturer, is not guaranteed or endorsed by the publisher.
